# Responsibility and cooperativeness are constrained, not determined

**DOI:** 10.3389/fpsyg.2014.00308

**Published:** 2014-05-07

**Authors:** Danilo Garcia, Alva Stråge, Sebastian Lundström, Susanna Radovic, Sven Brändström, Maria Råstam, Thomas Nilsson, C. Robert Cloninger, Nóra Kerekes, Henrik Anckarsäter

**Affiliations:** ^1^Institute of Neuroscience and Physiology, Centre for Ethics, Law and Mental Health, University of GothenburgGothenburg, Sweden; ^2^Network for Empowerment and Well-BeingGothenburg, Sweden; ^3^Department of Philosophy, Linguistics and Theory of Science, University of GothenburgGothenburg, Sweden; ^4^Swedish Prison and Probation Service, R&D UnitGothenburg, Sweden; ^5^Institute of Neuroscience and Physiology, Gillberg Neuropsychiatry Centre, University of GothenburgGothenburg, Sweden; ^6^Department of Clinical Sciences, Lund UniversityLund, Sweden; ^7^Department of Psychiatry and Genetics, Washington University School of Medicine in St. LouisSt. Louis, MO, USA; ^8^Department of Clinical Sciences, Lund UniversityMalmö, Sweden

**Keywords:** personality, self-directedness, agency, communion, cooperativeness, free will, determinism, temperament and character inventory

In the last decades, voices from the scientific community have advocated rejection of free will and personal responsibility (e.g., Cashmore, 2010). Interpretations of findings from neuroscience from the last decades have been used to support this deterministic assumption. Recent experiments, for example, have detected brain activity in the prefrontal and parietal cortex up to 10 s before the person is aware of any decision-making process (Soon et al., 2008; see also Libet et al., 1983). This has been interpreted as suggesting that consciousness is a *post-hoc* phenomenon, caused by unconscious neural activity in the brain. The view of the physical world as determined, and the human brain as the organ that enables the mind, leads to the conclusion that brains and minds are both determined. If free will is an illusion, the ramifications to penal law and personal responsibility need to be reconsidered (Cheema and Virk, 2012). Nevertheless, as humans we have an innate urge to experience a sense of agency or responsibility for our actions (Nichols, 2011). Even when confronted with setbacks, disappointments, and failures, humans maintain a sense of personal responsibility (see Gazzaniga, 2011). Failing to be aware of the self as the cause of one's own actions leads to aggressive and less helpful behavior (Baumeister et al., 2009). This article presents evidence that there is a possibility to develop an adequate sense of responsibility and cooperation in the presence of genetic and environmental adversity.

We used data from a population-based cohort of 15-year old twin pairs, to assess genetic and environmental impact on self-reported Self-directedness and Cooperativeness by classic twin methodology (this data was published in Garcia et al., 2013). Here, however, we also describe the variation of these character traits in monozygotic vs. dizygotic co-twins of individuals with extremely low scores in Self-directedness and Cooperativeness. Self-directedness indicates how responsible, purposeful, reliable and resourceful an individual is in working to achieve her goals and values (i.e., agency), while Cooperativeness indicates how well adapted she is in getting along with others fairly, flexibly and with kindness (i.e., communion). Self-directedness and Cooperativeness are highly predictive of mental health problems across diagnostic categories (Cloninger et al., 1993).

The participants consist of same-sex twins included in the Child and Adolescent Twin Study in Sweden (CATSS), which is a nation-wide, population based, longitudinal twin study of mental health. Currently, the CATSS includes around 20,000 twins, born from 1992 to 2002, and has a response rate of about 80% (Anckarsäter et al., 2011), which makes it the world's largest child- and adolescent psychiatric twin study. At the age of 15 the CATSS-twins of the 1994–1995 birth cohorts completed the short (125 items) or the longer version (238 items) of the Temperament and Character Inventory (TCI; Cloninger et al., 1993). The overall TCI response rate was about 55% and the total number of participants was 2714 (369 of whom completed the longer TCI as part of a clinical study). Zygosity is determined by a validated algorithm with a >95% predictive value compared to DNA-testing (Hannelius et al., 2007). In order to use all TCI responses, we extracted the items from the 238-version that correspond to the 125-version (see Garcia et al., 2013). Mx and SAS (version 9.3) softwares were used to disentangle the genetic and environmental contribution of Self-directedness and Cooperativeness. Intra-class correlations and univariate genetic analyses, by structural equation modeling, were calculated on the continuous scores of each dimension separately. A total of 831 adolescent same-sex twin pairs (423 monozygotic pairs and 408 dizygotic pairs) were used in this specific analysis.

We used the TCI mean and standard deviation scores of the 1994-CATSS cohort (*n* = 1340) in order to standardize the twins' Self-directedness and Cooperativeness scores to *t*-scores (IBM SPSS version 19) and identified the probands whose *t*-scores were ≤2 standard deviations below the general population's mean in each character dimension. If both twins in a pair met this criterion, one of them was randomly identified as proband and the other as co-twin. We found 28 monozygotic-twins and 32 dizygotic-twins who showed extremely low Self-directedness and 25 monozygotic-twins and 22 dizygotic-twins who showed extremely low Cooperativeness. In order to not depend on self-assessments alone, co-twins to probands who also had a parent rated DSM-IV disruptive behavior disorder (i.e., attention-deficit/hyperactivity disorder, oppositional defiant disorder or conduct disorder according to parent interviews by the Autism—Tics, AD/HD and other Comorbidities Inventory; Hansson et al., 2005) were specifically indicated in the plots. Twenty-nine percent of the variance in Self-directedness was attributable to genetic factors, 22% to environmental factors that make the twins reared together similar, and 49% to environmental factors that make the twins dissimilar. Thirty-eight percent of Cooperativeness was attributable to genetic factors, 21% to environmental factors that make twins similar, and 41% to environmental factors that make twins dissimilar (see Garcia et al., 2013).

Both monozygotic and dizygotic co-twins of probands with extremely low Self-directedness and Cooperativeness had an increased probability of reporting low (≤1 standard deviations) or extremely low Self-directedness and Cooperativeness (≤2 standard deviations) as compared to the general population (see Figure 1, population mean = 50, standard deviation = 10), but a considerable number of these individuals had developed character in the average or high range, in spite of having exactly the same, or half the genetic susceptibility of the problem-laden individuals. Co-twins to probands with both self-reported and objectively observed problems did not differ from the overall pattern.

**Figure 1:**
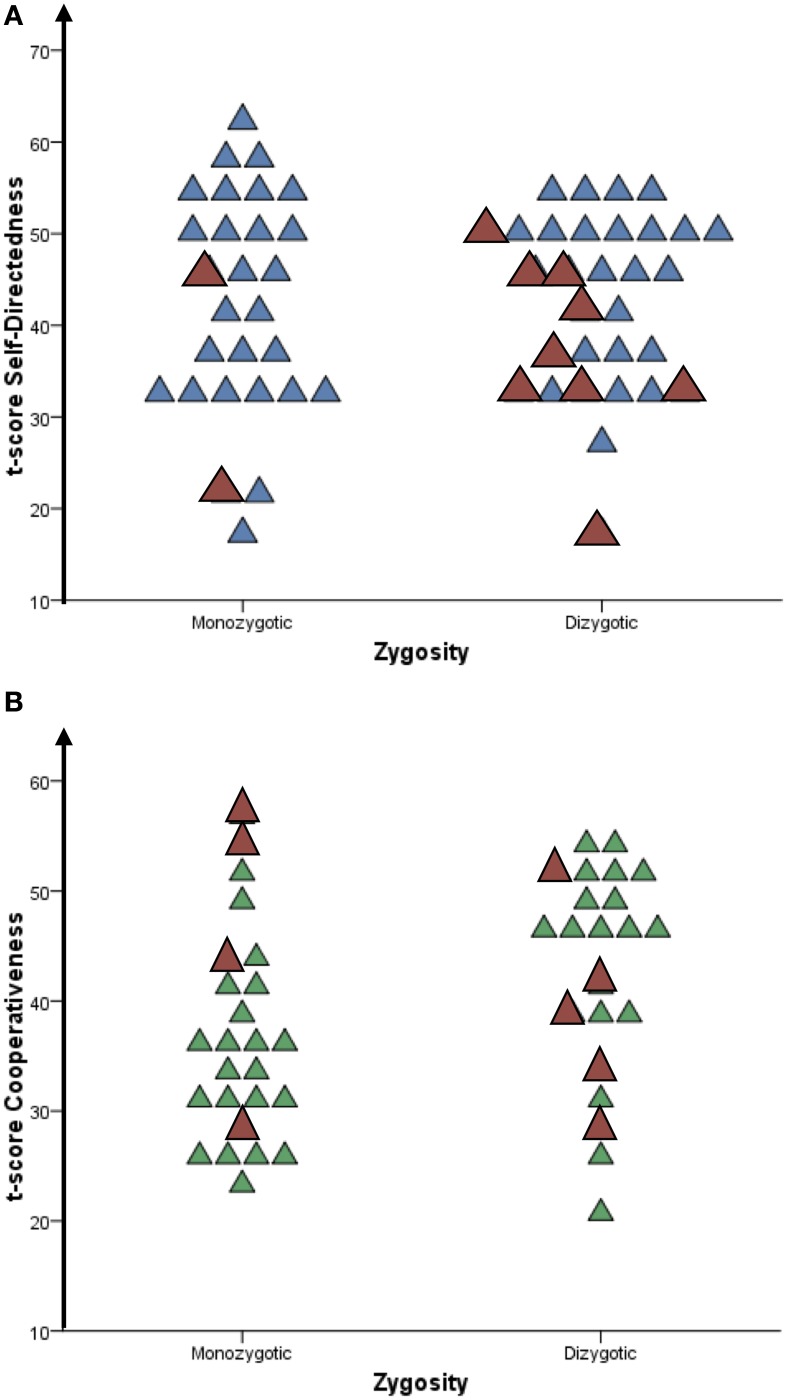
**(A)** Distribution of Self-directedness (*t*-scores) in monozygotic and dizygotic co-twins of adolescents with extremely low Self-directedness (<2 SD). **(B)** Distribution of Cooperativeness (*t*-scores) in MZ and DZ co-twins of adolescents with extremely low Cooperativeness (<2 SD). Each triangle denotes one individual. 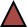 = Co-twin has a disruptive behavior disorder.

Environmental and genetic adversities thus clearly give unequal opportunities to develop a sense of self-control, cooperation, and responsibility, but with a substantial plasticity. This study shows that it is possible to develop a mature character in spite of a genetic make-up and/or early environment associated with a deficient sense of responsibility and cooperation. A self-reported sense of responsibility and cooperation may not reflect actual freedom of action, but the scales used here were developed to assess the degree to which a person experiences that her behaviors over the life-span actually converge with her intentions, desires and conceptualized goals. The scales have also been shown to measure constructs that are highly relevant for mental health, including pro-social and constructive behavior patterns (Cloninger, 2004).

## Conclusion and final remarks

The study does not support suggestions that society should change its stance/attitude on personal and penal responsibility in view of genetic or environmental determinism, or that interventions meant to promote responsibility and cooperation would be meaningless. Furthermore, philosophers have different ideas about what it takes to say that we humans have free will in a moral-responsibility-grounding sense (see Jeppsson, 2012). In contrast to the neuroscience-determinism outlined in the beginning of this article (i.e., that unconscious neural activity in the brain enables the mind), philosophers also refer to determinism in terms of that, given the laws of nature and the past being precisely what they are, only one future is possible. In other words, if two persons had the exact same genetic structure and were exposed to exactly the same environmental influences, they would behave exactly the same. In this framework, some philosophers argue that humans cannot be free or morally responsible for anything if the world is deterministic or nearly deterministic. Others argue that, as long as we can choose what to do based on reasons and have enough self-control; humans can be considered free and morally responsible (e.g., Lenman, 2006). In this article we have showed that people do not lack moral responsibility only because they have “bad genes” or come from a “bad environment.” Furthermore, the results shows that so far, we have no proof that determinism in the wider sense is true. However, neither does our study disprove that the world is deterministic in this sense. Twin studies cannot falsify this thesis, since not even twins growing up together are ever exposed to the same environmental influences down to the last detail.

### Conflict of interest statement

The authors declare that the research was conducted in the absence of any commercial or financial relationships that could be construed as a potential conflict of interest.

## References

[B1] Anckarsäter H. Lundström S. Kollberg L. Kerekes N. Palm C. Carlström E. (2011). The Child and Adolescent Twin Study in Sweden (CATSS). Twin Res. Hum. Genet. 14, 495–508 10.1375/twin.14.6.495 22506305

[B2] Baumeister R. F. Masicampo E. J. DeWall C. N. (2009). Prosocial benefits of feeling free: disbelief in free will increases aggression and reduces helpfulness. Pers. Soc. Psychol. Bull. 35, 206–268 10.1177/0146167208327217 19141628

[B3] Cashmore A. R. (2010). The Lucretian swerve: the biological basis of human behavior and the criminal justice system. Proc. Natl. Acad. Sci. U.S.A. 107, 4499–4504 10.1073/pnas.0915161107 20142481 PMC2842067

[B4] Cheema A. A. Virk A. (2012). Reinventing Lombroso in the era of genetic revolution: whether criminal justice system actually imparts justice or is based on “convenience of assumption?” Int. J. Criminol. Sociol. Theory 5, 936–946

[B5] Cloninger C. R. (2004). Feeling Good: The Science of Wellbeing. New York, NY: Oxford University Press

[B6] Cloninger C. R. Svrakic D. M. Przybeck T. R. (1993). A psychobiological model of temperament and character. Arch. Gen. Psychiatry 50, 975–990 10.1001/archpsyc.1993.01820240059008 8250684

[B7] Garcia D. Lundström S. Brändström S. Råstam M. Cloninger C. R. Kerekes N. (2013). Temperament and Character in the Child and Adolescent Twin Study in Sweden (CATSS): comparison to the general population, and genetic structure analysis. PLoS ONE 8:e70475 10.1371/journal.pone.0070475 23940581 PMC3734246

[B8] Gazzaniga M. S. (2011). Who's in Charge? Free will and the Science of the Brain. New York, NY: HarperCollins Publishers

[B9] Hannelius U. Gherman L. Mäkelä V. Lindstedt A. Zucchelli M. Lagerberg C. (2007). Large-scale zygosity testing using single nucleotide polymorphisms. Twin Res. Hum. Genet. 10, 604–625 10.1375/twin.10.4.604 17708702

[B10] Hansson S. L. Svanström-Rojvall A. Råstam M. Gillberg I. C. Gillberg C. Anckarsater H. (2005). Psychiatric telephone interview with parents for screening of childhood autism-tics, attention-deficit hyperactivity disorder and other comorbidities (A-TAC): preliminary reliability and validity. Br. J. Psychiatry 187, 262–267 10.1192/bjp.187.3.262 16135864

[B11] Jeppsson S. (2012). Practical Perspective Compatibilism. Doctoral Dissertation, Stockholm University.

[B12] Lenman J. (2006). Compatibilism and contractualism: the possibility of moral responsibility. Ethics 117, 7–31 10.1086/508035

[B13] Libet B. Gleason C. A. Wright E. W. Pearl D. K. (1983). Time of conscious intention to act in relation to onset of cerebral activity (readiness-potential). The unconscious initiation of a freely voluntary act. Brain 106, 623–642 6640273 10.1093/brain/106.3.623

[B14] Nichols S. (2011). Experimental philosophy and the problem of free will. Science 331, 1401–1403 10.1126/science.1192931 21415346

[B15] Soon C. S. Brass M. Heinze H. J. Haynes J. D. (2008). Unconscious determinants of free decisions in the human brain. Nat. Neurosci. 11, 543–545 10.1038/nn.2112 18408715

